# *hTERT* Gene Expression and Athlete’s Heart: A Study in Middle-Aged Endurance Athletes

**DOI:** 10.3390/genes16091104

**Published:** 2025-09-18

**Authors:** Caglar Ozmen, Nihal Inandiklioglu, Ozgur Gunasti, Hatice Rahimova, Omer Tepe, Rabia Eker Akilli, Pinar Ozmen Yildiz, Sanli Sadi Kurdak, Mustafa Demirtas

**Affiliations:** 1 Department of Cardiology, Faculty of Medicine, Çukurova University, Adana 01330, Türkiye; rahimli.89@mail.ru (H.R.); rabiaekerakilli@gmail.com (R.E.A.); drmustafademirtas@gmail.com (M.D.); 2 Department of Medical Biology, Faculty of Medicine, Yozgat Bozok University, Yozgat 66900, Türkiye; nihal.inandiklioglu@yobu.edu.tr; 3 Department of Physiology, Faculty of Medicine, Çukurova University, Adana 01330, Türkiye; ogunasti@cu.edu.tr (O.G.); sskurdak@cu.edu.tr (S.S.K.); 4 Department of Cardiology, Osmaniye State Hospital, Osmaniye 80000, Türkiye; omer.tepe0133@gmail.com; 5 Department of Cardiology, Health Sciences University, Adana City Training and Research Hospital, Adana 01270, Türkiye; pinarymnn@hotmail.com

**Keywords:** athlete, cardiology, hTERT, telomerase, VO_2_peak

## Abstract

**Background/Objectives:** Telomeres and the enzyme telomerase play essential roles in cellular aging and cardiovascular health. Physical activity is thought to influence telomere dynamics via upregulation of the *hTERT* gene, which encodes the catalytic subunit of telomerase. However, data on this relationship in middle-aged endurance athletes remain limited. This study aimed to investigate the association between long-term endurance training, cardiac structural adaptations, and *hTERT* gene expression in middle-aged elite athletes. **Methods:** A total of 38 middle-aged elite runners and 37 age-matched sedentary controls were enrolled. Echocardiographic assessments, VO_2_peak measurements, and *hTERT* gene expression analysis using RT-PCR were conducted. Left ventricular mass (LVM), wall thicknesses, and cardiac volumes were compared, and correlations with *hTERT* expression were analyzed. **Results:** Athletes demonstrated significantly higher VO_2_peak and echocardiographic parameters including LVEDD, LV mass, and wall thicknesses (*p* < 0.05). *hTERT* gene expression was 2.06-fold higher in athletes compared to controls. Significant positive correlations were observed between *hTERT* expression and VO_2_peak, LVM, LV wall thicknesses, and right ventricular parameters. **Conclusions:** These findings suggest that regular aerobic exercise may contribute to both improved cardiovascular performance and cellular longevity by enhancing telomerase-related mechanisms.

## 1. Introduction

Telomeres are specialized heterochromatic regions located at the ends of chromosomes in eukaryotic organisms, composed of repeated DNA sequences (TTAGGG). The enzyme telomerase is a ribonucleoprotein complex with reverse transcriptase activity that functions as a specialized DNA polymerase responsible for the synthesis of these telomeric repeats. Telomerase, a large enzyme complex, synthesizes telomeric DNA on one strand and plays a crucial role in maintaining chromosome integrity [1]. Various environmental factors, including lifestyle, diet, physical activity, alcohol and tobacco consumption, oxidative stress, and the presence of certain antioxidants, are known to influence telomere length and telomerase activity. Telomere shortening has been implicated in atherogenesis and exerts detrimental effects on cardiovascular repair mechanisms [2]. Studies support that telomere shortening-induced endothelial dysfunction and replicative senescence play a critical role in coronary atherogenesis, myocardial infarction, and ischemic heart disease [3].

Regular physical exercise induces several electrophysiological and morphological changes in the heart, a condition known as “athlete’s heart.” In such individuals, increases in left ventricular mass (LVM), chamber dimensions, and wall thickness are commonly observed. Athletes with left ventricular hypertrophy (LVH) typically maintain a normal ejection fraction (EF) and do not exhibit signs of systolic or diastolic dysfunction [4,5]. However, during intense endurance training, transient reductions in right ventricular (RV) function have also been reported [6]. Increased LVM and cardiac dimensions are considered key indicators of target organ damage resulting from lifelong exposure to elevated blood pressure and other vascular risk factors. Higher LVM has been associated with an increased risk of clinical cardiovascular disease (CVD) [7]. In endurance sports involving regular and intensive physical training, physiological increases in left ventricular wall thickness, chamber size, and mass have been documented [8]. In contrast, sports requiring high levels of muscular strength tend to increase LVM without significantly altering chamber diameters [9]. The definition of the upper physiological limits of hypertrophy seen in athlete’s heart is largely based on echocardiographic studies conducted in adult athletes [10].

Physical inactivity and lack of exercise are thought to decrease telomerase expression, potentially negatively affecting overall health, healthy aging, and longevity [11]. In contrast, consistent engagement in endurance-based physical activities (e.g., running) has been shown to preserve telomere length throughout the human lifespan [12]. Endurance athletes have been shown to exhibit increased leukocyte TERT/telomerase activity compared to inactive controls, suggesting that exercise training may preserve telomeres through telomerase upregulation and thereby support healthy biological aging [11]. As a physiological stressor, exercise has been shown to upregulate *hTERT* mRNA expression and stimulate telomerase enzyme activity. One of the core variables in exercise physiology is maximal oxygen consumption (VO_2_peak), which is commonly used to measure an individual’s aerobic capacity and cardiorespiratory fitness [13]. It has been reported that genetic factors account for approximately 40% of VO_2_peak variation, that VO_2_peak declines with age, is generally lower in females compared to males, and is also influenced by body mass index and activity level [14]. Previous studies have demonstrated that individuals who engage in regular physical exercise exhibit longer telomere lengths and higher telomerase activity compared to their sedentary counterparts. However, regular exercise is thought to protect against age-related telomere shortening, as older adults who engage in endurance training have been found to have telomere lengths similar to those of younger adults [15]. Researchers have demonstrated that the beneficial effects of endurance exercise training on leukocyte telomere maintenance are associated with a higher VO_2_peak and a lower resting heart rate, and they have also shown a plateauing effect between weekly running and cycling distance and the increase in leukocyte telomere length [16]. Furthermore, physical activity has been shown to modulate telomere-stabilizing proteins and protects against stress-induced vascular apoptosis in mice and humans [17].

In this study, we aimed to compare echocardiographic findings, VO_2_peak capacity, and the expression of the *hTERT* gene—which encodes the catalytic subunit of telomerase enzymes responsible for telomere maintenance—between middle-aged elite athletes and healthy sedentary controls. By doing so, we evaluated the potential effects of sustained physical activity on cardiac health and telomerase-related cellular aging in middle-aged elite athletes.

## 2. Materials and Methods

### 2.1. Study Population

This study was conducted at the Department of Cardiology, Çukurova University, involving 38 middle-aged male elite runners and 37 healthy male sedentary individuals. The athlete group consisted of individuals aged 40 years and older who engaged in regular recreational running and participated in half-marathons and marathons. All participants were healthy individuals without a history of diabetes, cardiovascular or renal disease, and were not using any hormonal or other medications. The control group consisted of age-matched healthy individuals who did not engage in regular exercise. The inclusion criteria were training regularly for at least 2 years at one of the running clubs in our region and prior participation in at least 1 half marathon or a marathon. Any history of chronic disease, drug use, dietary supplements, or supplement usage was accepted as exclusion criteria. In addition, even if the participant stated that they exercised, a VO_2_peak value below 35 mL/kg/min in the cardiopulmonary exercise test was also accepted as an exclusion criterion.

### 2.2. Genetic Analysis

To detect *hTERT* mRNA expression, total RNA was isolated from peripheral blood samples using the High Pure RNA Isolation Kit (Roche, Sandhofer Strasse 116, 68305 Mannheim, Germany), following the manufacturer’s protocol. The obtained RNA samples were reverse transcribed into cDNA using the Transcriptor First Strand cDNA Synthesis Kit (Roche, Germany), and after incubation, the cDNA samples were stored at −20 °C. cDNA concentrations were measured using a fluorometer (QFX, 3411 Silverside Road, 100 Hagley Building, Wilmington, DE 19810, USA), and all samples were standardized to a concentration of 10 ng/µL. *hTERT* (Thermo, cat. no: Hs00972650_m1) gene expression analysis was performed using TaqMan Gene Expression Assays (Thermo Fisher Scientific, 168 Third Avenue, Waltham, MA 02451, USA) in a real-time PCR system (CFX96, BioRad, 1000 Alfred Nobel Drive, Hercules, CA 94547, USA). Beta-actin (*ACTB*) was used as the internal control gene. cDNA samples were studied 3 times under the same conditions. The mean of these three measurements was used in the analyses. The quantification cycle (Ct) for each sample was recorded, and relative expression levels were analyzed using the 2^∆∆Ct^ method.

### 2.3. Cardiological Assessment

All participants underwent echocardiographic evaluation using a transthoracic echocardiography system. Standard imaging planes were used to acquire cardiac images. Participants were positioned in the left lateral decubitus position, and measurements were obtained using 2D, M-mode, color Doppler, and pulsed wave Doppler through parasternal long and short axes and apical four- and five-chamber views. Structural and functional cardiac assessments were performed in accordance with the current literature and consensus guidelines. Echocardiographic measurements followed the recommendations of the American Society of Echocardiography and the European Association of Cardiovascular Imaging. The left ventricular end-systolic diameter (ESD), end-diastolic diameter (EDD), interventricular septum thickness (IVS), left ventricular posterior wall thickness (LVPW), and anteroposterior left atrial dimension were measured in the parasternal long axis view. Left atrial volume was calculated using the “prolate ellipsoid” method, incorporating anteroposterior, vertical, and horizontal dimensions obtained from the parasternal long axis and apical four-chamber views [18]. The end-diastolic internal dimensions and wall thicknesses were obtained using M-mode measurements averaged over at least three cardiac cycles, following the leading-edge method as recommended by the American Society of Echocardiography [19]. Left ventricular mass (LVM) was calculated using the formula [20]:

LVM = 0.8 × [1.04 × ((LVDD + LVWT)^3^ − (LVDD)^3^)] + 0.6

### 2.4. Exercise Physiology Assessment

Measurements were taken in the morning at consistent times by the same examiner. Body weight was measured barefoot using a scale with a precision of 0.01 kg, and height was measured in a standing position using a stadiometer with 0.01 cm precision. The athletic group underwent a maximal cardiopulmonary exercise test to assess performance capacity. Tests were completed during the non-competition period while the participants continued their regular exercise routine. During this test, a face mask was used to measure inspired oxygen and expired carbon dioxide fractions on a breath-by-breath basis using an indirect calorimeter device (PFT COSMED). The treadmill (HP Cosmos) test began at a 1% incline and a speed of 6 km/h, with incremental increases of 1 km/h every minute. Heart rate was recorded simultaneously. The test was terminated upon reaching any of the following criteria: achieving 90% of predicted maximal heart rate, a plateau in oxygen uptake, a respiratory quotient (RQ) > 1.15, or voluntary exhaustion [21]. For individuals self-reporting as athletes, a peak oxygen consumption (peak VO_2_) value exceeding 35 mL/kg/min confirmed their athletic status based on the maximal cardiopulmonary exercise test [22,23,24,25]. For sedentary individuals, peak VO_2_ values were estimated using the formula described by Davis et al. [26].

### 2.5. Statistical Analysis

Statistical analyses were conducted using SPSS software (Version 20.0, SPSS Inc., Chicago, IL, USA). Continuous variables were tested for normality using the Kolmogorov–Smirnov and Shapiro–Wilk tests. Normally distributed variables were presented as mean ± standard deviation (*p* > 0.05), while non-normally distributed variables were expressed as medians. Categorical variables were analyzed using the Chi-square test or Fisher’s exact test. Inter-group comparisons were performed using Student’s *t*-test for normally distributed data and the Mann–Whitney U test for non-normally distributed data. A *p*-value of <0.05 was considered statistically significant.

## 3. Results

A total of 75 participants were included in the study, categorized into athlete and control groups. Demographic and clinical data of the athlete and control groups were statistically compared (Table 1). The mean age of the athlete group was 46.5 ± 8.1 years, while it was 44.11 ± 5.51 years in the control group. No statistically significant difference was observed between the groups in terms of age (*p* > 0.05).

When cardiological parameters were evaluated, all echocardiographic parameters except for the right ventricular end-diastolic diameter (RVEDD) showed statistically significant differences in favor of the athlete group (*p* < 0.05). In our study, elite athletes demonstrated significantly greater left ventricular end-diastolic diameter (LVEDD), left ventricular mass (LV mass), left ventricular end-diastolic volume (LVEDV), right ventricular wall thickness (RV wall thickness), RVEDD, left ventricular septal wall thickness (LV septal wall thickness), and left ventricular posterior wall thickness (LV posterior wall thickness) compared to the non-athletic healthy individuals. The LV septal wall thickness in elite athletes ranged from 7 mm to 14 mm. Among the 38 elite athletes, 5 individuals (13%) exhibited LV septal wall thickness values that exceeded the predicted upper physiological limit (≥12 mm). In these five individuals, absolute LV wall thickness (LVWT) measurements ranged between 12 mm and 14 mm. In laboratory assessments comparing elite athletes with non-athletic healthy individuals, glucose and total cholesterol levels were found to be significantly higher in the elite athlete group. Regarding physiological performance, VO_2_peak levels were significantly higher in athletes compared to the control group (*p* < 0.05).

Expression analysis results of the *hTERT* gene in middle-aged elite athletes were statistically compared with those of the control group (Table 2). *hTERT* gene expression levels in the athlete group were found to be 2.06-fold higher than those in the control group. Correlation analysis revealed a statistically significant positive correlation between *hTERT* gene expression and VO_2_peak as well as between VO_2_peak and RV thickness (Table 3).

## 4. Discussion

Telomere maintenance and stabilization are primarily regulated by the enzyme telomerase [27]. An increase in telomerase activity not only preserves telomere length but also supports healthy cellular functions and long-term immune competence [28]. Several studies investigating telomere length and telomerase activity in relation to cardiovascular diseases have demonstrated that individuals with shorter telomeres are at higher cardiovascular risk [2,29]. Besides that, significant improvements in ventricular function have been observed with telomerase gene transfer therapy in animal models [30]. A sedentary lifestyle has been shown to negatively impact telomere dynamics. Recent studies have demonstrated a positive correlation between leukocyte telomere length and physical activity in healthy individuals. It has been reported that physically active individuals (engaging in ≥6 h of exercise per week) exhibit leukocyte telomeres that are, on average, 151 nucleotides longer than those of inactive individuals, after adjusting for age and sex [31]. In another study comparing leukocyte telomere length, VO_2_peak, and physical activity levels, both young and older adults who performed regular aerobic exercise had longer telomeres and higher VO_2_peak levels compared to their sedentary peers [32]. While evidence has demonstrated that aerobic endurance and high-intensity interval training can enhance telomerase activity and telomere length in peripheral blood mononuclear cells, one study indicated that alterations in telomere length were not necessarily correlated with variations in cardiorespiratory fitness as defined by VO_2_peak [33]. In a study involving individuals aged 50–70 years, moderate degrees of physical activity were shown to exert a protective influence on leukocyte telomere length, and a positive association was identified between telomere length and telomerase activity [12]. Denham et al. reported a twofold-upregulation of TERT (2.0-fold) mRNA expression in endurance athletes compared to healthy controls. Furthermore, these levels were found to be associated with a lower resting heart rate and superior VO_2_peak [11]. In our study, *hTERT* gene expression was found to be 2.06-fold higher in middle-aged elite athletes compared to sedentary individuals. When our findings are compared with those in the existing literature, they are consistent with previous studies [11,16], further supporting the positive impact of regular physical activity on telomerase expression. In line with the exposome perspective, the recent literature underscores the importance of integrating genetic, molecular, and environmental determinants to better understand health and longevity in athletes. Spanakis et al. emphasized that combining conventional biochemical and physiological assessments with advanced biomedical tools such as telomere analysis, genotyping/phenotypic profiling, and metabolomics provides a multidimensional framework for evaluating athletic performance and biological aging [34]. Complementary to this, Baliou et al. highlighted that exercise exerts protective effects on telomere dynamics through the reduction in oxidative stress and inflammation, alongside enhanced telomerase activity, thereby mitigating cellular aging processes in athletes [35]. Furthermore, Penggalih et al. argued that omics-based precision nutrition enables the development of personalized dietary strategies aligned with athletes’ genetic and metabolic profiles, supporting recovery, injury prevention, and performance optimization [36]. Collectively, these findings indicate that adopting an exposome-oriented approach-linking genotype/phenotype data with lifestyle and environmental factors- may be essential for identifying determinants of both health and longevity in athletic populations.

In our study, we found that intense and regular exercise induces changes in cardiac structure consistent with athlete’s heart, as evaluated by echocardiography. This adaptive response is considered a crucial component of athletic performance, enabling the heart to maintain elevated cardiac output in response to increased muscle demand during prolonged exercise. In the elite athlete group, we observed significant increases in left ventricular end-diastolic diameter (LVEDD), left ventricular mass (LV mass), left ventricular end-diastolic volume (LVEDV), right ventricular wall thickness, right ventricular end-diastolic diameter (RVEDD), left ventricular septal wall thickness, and left ventricular posterior wall thickness compared to the control group. In structurally normal athletic hearts without any systemic or cardiac disease, chronic and repetitive intense exercise leads to enlargement of cardiac chambers, increased LV wall thickness, and elevated LV mass. Factors such as age, hypertension, insulin resistance, and obesity—each of which are positively associated with LV mass—have been reported to be inversely associated with *hTERT* gene expression [37,38,39,40]. Thus, we initially hypothesized an inverse relationship between LV mass and *hTERT* gene expression. However, surprisingly, in our study, we did not observe an association between LVmass and *hTERT* gene expression. We were also unable to detect any correlation between *hTERT* gene expression and LV septal wall thickness or LV posterior wall thickness.

This study presents several limitations that must be acknowledged when interpreting the results. Firstly, the study includes a comparatively small sample size. Although statistically adequate, the sample size was relatively limited, which may restrict the generalizability of the results to wider populations. Secondly, *hTERT* gene expression activity and cardiac changes were assessed at a single time point. A longitudinal design would provide more robust information on the dynamics of these changes over time. Another limitation of our study is the absence of cardiopulmonary exercise testing in the control group.

## 5. Conclusions

In conclusion, our findings suggest that long-term and intensive physical activity in middle-aged elite athletes is associated with significant physiological cardiac remodeling, characterized by increased left ventricular mass, chamber dimensions, and wall thickness—all consistent with the athlete’s heart phenotype. Additionally, a marked upregulation of *hTERT* gene expression was observed in the athlete group. Importantly, *hTERT* expression showed a positive correlation with VO_2_peak, suggesting a potential mechanistic link between cardiorespiratory fitness and telomere regulation. Although no association was found between *hTERT* expression and specific structural parameters such as LV mass, septal wall thickness, or posterior wall thickness, the elevated *hTERT* levels may reflect broader cellular adaptations induced by regular exercise. These findings support the notion that aerobic training may not only enhance cardiovascular performance but also promote genomic stability and cellular longevity through telomerase-related pathways. Future longitudinal and mechanistic studies are warranted to further elucidate the role of telomerase in exercise-induced cardiac and systemic remodeling.

## Figures and Tables

**Table 1:** Comparison of clinical and biochemical parameters in athlete and control groups.

	Athlete (*n* = 38)	Control (*n* = 37)	*p* Value
Age (year)	46.5 ± 8.1	44.11 ± 5.51	0.097
Size (cm)	171.3 ± 6.5	171.3 ± 6.1	0.99
Weight (kg)	69.9 ± 5.1	67.7 ± 7.9	0.060
BMI	23.6 ± 1.1	22.9 ± 1.9	0.066
RV Thickness	3.4 ± 0.8	0.8 ± 1.2	0.000
EF	69.5 ± 3.8	66.8 ± 3.5	0.002
IVS	10.0 ± 1.3	9.2 ± 1.3	0.007
PW	9.7 ± 0.8	8.6 ± 1.0	0.000
LVEDD	49.0 ± 3.5	46.9 ± 5.1	0.041
LVM	95.0 ± 17.8	76.7 ± 14.7	0.000
LVEDV	129.8 ± 21.9	79.0 ± 7.7	0.000
RVEDD	27.8 ± 4.4	27.6 ± 4.4	0.787
Sports age (year)	10.6 ± 7.4	0.0 ± 0.0	0.000
Training volume (km/week)	56.4 ± 5.5	0.0 ± 0.0	0.000
VO_2_peak (mL/kg/min)	44.8 ± 5.5	32.2 ± 3.9	0.000
Creatinine	0.7 ± 0.2	0.7 ± 0.2	0.117
Glucose	98.9 ± 5.1	92.9 ± 4.8	0.000
HDL	54.2 ± 11.7	51.4 ± 10.9	0.287
LDL	107.8 ± 22.1	103.6 ± 25.5	0.452
Total cholesterol *	231.4 ± 27.8	213.5 ± 28.9	0.008

RV: right ventricular; EF: ejection fraction; LVEDD: left ventricular end-diastolic diameter; LVM: left ventricular mass; IVS: interventricular septum; PW: posterior wall; LVEDV: left ventricular end-diastolic volume; RVEDD: right ventricular end-diastolic diameter; HDL: high-density lipoprotein; LDL: low-density lipoprotein. Values are presented as mean ± standard deviation. *p* < 0.05 is significant.

**Table 2:** *hTERT* gene expression analysis result.

Gene Symbol	AVG ΔCt		2^−ΔCt^		Fold Change *	*p* Value **	Fold Up- or Down Regulation
	Athlete Group	Control Group	Athlete Group	Control Group	Athlete/Control		Athlete/Control
*ACTB*	0.00	0.00	1.000000	1.000000	1.00	nan	1.00
*hTERT*	12.76	13.80	0.000145	0.000070	2.06	0.0013	2.06

* Fold-Change (2^(−ΔΔCT)^) is the normalized gene expression in Test Sample (2^(−ΔCT)^) divided by the normalized gene expression in Control Sample (2^(−ΔCT)^). ** *p* values are calculated according to Student’s *t*-test of repeated 2^(−ΔCT)^ values for each gene in the control group and treatment groups, and *p* values less than 0.05 are shown.

**Table 3:** Correlation of *hTERT* with cardiological parameters.

	RV Thickness	EF	IVS	PW	LVEDD	LVM	LVEDV	VO_2_peak
RV Thickness	1.000							
**EF**	−0.252	1.000						
**IVS**	−0.108	−0.126	1.000					
**PW**	−0.046	−0.033	0.625 **	1.000				
**LVEDD**	0.210	−0.353 *	0.027	0.122	1.000			
**LVM**	−0.032	−0.084	−0.147	−0.244	0.129	1.000		
**LVEDV**	−0.253	0.143	0.282	0.469 **	−0.091	0.024	1.000	
**VO_2_peak**	0.472 **	−0.054	0.112	0.114	−0.007	−0.146	0.066	1.000
***hTERT***	0.133	−0.202	0.135	−0.038	0.070	−0.221	0.141	0.341 *

Spearman’s rho test. ** Correlation is significant at the 0.01 level (2-tailed). * Correlation is significant at the 0.05 level (2-tailed).

## Data Availability

The original contributions presented in this study are included in the article. Further inquiries can be directed to the corresponding authors.

## References

[1] Blackburn E.H., Epel E.S., Lin J. (2015). Human telomere biology: A contributory and interactive factor in aging, disease risks, and protection. Science.

[2] Yeh J.K., Wang C.Y. (2016). Telomeres and telomerase in cardiovascular diseases. Genes.

[3] Minamino T., Miyauchi H., Yoshida T., Ishida Y., Yoshida H., Komuro I. (2002). Endothelial cell senescence in human atherosclerosis: Role of telomere in endothelial dysfunction. Circulation.

[4] La Gerche A., Taylor A.J., Prior D.L. (2009). Athlete’s heart: The potential for multimodality imaging to address the critical remaining questions. JACC Cardiovasc. Imaging.

[5] Lazzeroni D., Rimoldi O., Camici P.G. (2016). From left ventricular hypertrophy to dysfunction and failure. Circ. J..

[6] D’Andrea A., La Gerche A., Golia E., Padalino R., Calabrò R., Russo M.G., Bossone E. (2015). Physiologic and pathophysiologic changes in the right heart in highly trained athletes. Herz.

[7] Devereux R.B., Koren M.J., de Simone G., Roman M.J., Laragh J.H. (1992). Left ventricular mass as a measure of preclinical hypertensive disease. Am. J. Hypertens..

[8] Maron B.J. (1986). Structural features of the athlete’s heart as defined by echocardiogaphy. J. Am. Coll. Cardiol..

[9] Yilmaz M., Dagli M.N. (2016). Athlete Health and Exercise-Related Deaths. Fırat Üniversitesi Sağlık Bilim. Tıp Derg..

[10] Maron B.J., Isner J.M., McKenna W.J. (1994). 26th Bethesda conference: Recommendations for determining eligibility for competition in athletes with cardiovascular abnormalities. Task Force 3: Hypertrophic cardiomyopathy, myocarditis and other myopericardial diseases and mitral valve prolapse. J. Am. Coll. Cardiol..

[11] Denham J., Sellami M. (2021). Exercise training increases telomerase reverse transcriptase gene expression and telomerase activity: A systematic review and meta-analysis. Ageing Res. Rev..

[12] Ludlow A.T., Zimmerman J.B., Witkowski S., Hearn J.W., Hatfield B.D., Roth S.M. (2008). Relationship between physical activity level, telomere length, and telomerase activity. Med. Sci. Sports Exerc..

[13] Bassett D.R., Howley E.T. (2000). Limiting factors for maximum oxygen uptake and determinants of endurance performance. Med. Sci. Sports Exerc..

[14] Williams C.J., Williams M.G., Eynon N., Ashton K.J., Little J.P., Wisloff U., Coombes J.S. (2017). Genes to predict VO2max trainability: A systematic review. BMC Genom..

[15] Schellnegger M., Lin A.C., Hammer N., Kamolz L.P. (2022). Physical activity on telomere length as a biomarker for aging: A systematic review. Sports Med. Open.

[16] Denham J., O’Brien B.J., Prestes P.R., Brown N.J., Charchar F.J. (2016). Increased expression of telomere-regulating genes in endurance athletes with long leukocyte telomeres. J. Appl. Physiol..

[17] Werner C., Fürster T., Widmann T., Pöss J., Roggia C., Hanhoun M., Scharhag J., Büchner N., Meyer T., Kindermann W. (2009). Physical exercise prevents cellular senescence in circulating leukocytes and in the vessel wall. Circulation.

[18] Lang R.M., Bierig M., Devereux R.B., Flachskampf F.A., Foster E., Pellikka P.A., Picard M.H., Roman M.J., Seward J., Shanewise J.S. (2005). Recommendations for chamber quantification: A report from the American Society of Echocardiography’s Guidelines and Standards Committee and the Chamber Quantification Writing Group, developed in conjunction with the European Association of Echocardiography, a branch of the European Society of Cardiology. J. Am. Soc. Echocardiogr. Off. Publ. Am. Soc. Echocardiogr..

[19] Sahn D.J., DeMaria A., Kisslo J., Weyman A. (1978). Recommendations regarding quantitation in M-mode echocardiography: Results of a survey of echocardiographic measurements. Circulation.

[20] Devereux R.B., Alonso D.R., Lutas E.M., Gottlieb G.J., Campo E., Sachs I., Reichek N. (1986). Echocardiographic assessment of left ventricular hypertrophy: Comparison to necropsy findings. Am. J. Cardiol..

[21] American Thoracic Society/American College of Chest Physicians (2003). ATS/ACCP Statement on cardiopulmonary exercise testing. Am. J. Respir. Crit. Care Med..

[22] Clark A., De la Rosa A.B., DeRevere J.L., Astorino T.A. (2019). Effects of various interval training regimes on changes in maximal oxygen uptake, body composition, and muscular strength in sedentary women with obesity. Eur. J. Appl. Physiol..

[23] Jette M., Sidney K., Blumchen G. (1990). Metabolic equivalents (Mets) in exercise testing, exercise prescription, and evaluation of functional-capacity. Clin. Cardiol..

[24] Mendes M.A., da Silva I., Ramires V., Reichert F., Martins R., Ferreira R., Tomasi E. (2018). Metabolic equivalent of task (METs) thresholds as an indicator of physical activity intensity. PLoS ONE.

[25] Franklin B.A., Brinks J., Berra K., Lavie C.J., Gordon N.F., Sperling L.S. (2018). Using metabolic equivalents in clinical practice. Am. J. Cardiol..

[26] Davis J.A., Storer T.W., Caiozzo V.J., Pham P.H. (2002). Lower reference limit for maximal oxygen uptake in men and women. Clin. Physiol. Funct. Imaging.

[27] Förstemann K., Lingner J. (2005). Telomerase limits the extent of base pairing between template RNA and telomeric DNA. EMBO Rep..

[28] Effros R.B. (2011). Telomere/telomerase dynamics within the human immune system: Effect of chronic infection and stress. Exp. Gerontol..

[29] Pérez-Rivero G., Ruiz-Torres M.P., Rivas-Elena J.V., Jerkic M., Díez-Marques M.L., Lopez-Novoa J.M., Blasco M.A., Rodríguez-Puyol D. (2006). Mice deficient in telomerase activity develop hypertension because of an excess of endothelin production. Circulation.

[30] Zhan Y., Karlsson I.K., Karlsson R., Tillander A., Reynolds C.A., Pedersen N.L., Hägg S. (2017). Exploring the causal pathway from telomere length to coronary heart disease. Circ. Res..

[31] Cherkas L.F., Hunkin J.L., Kato B.S., Richards J.B. (2008). Association between physical activity in leisure time and leukocyte telomere length. Arch. Intern. Med..

[32] La Rocca T.J., Seals D.R., Pierce G. (2010). Leukocyte telomere length is preserved with aging in endurance exercise-trained adults and related to maximal aerobic capacity. Mech. Ageing Dev..

[33] Werner C.M., Hecksteden A., Morsch A., Zundler J., Wegmann M., Kratzsch J., Thiery J., Hohl M., Bittenbring J.T., Neumann F. (2019). Differential effects of endurance, interval, and resistance training on telomerase activity and telomere length in a randomized, controlled study. Eur. Heart J..

[34] Spanakis M., Fragkiadaki P., Renieri E., Vakonaki E., Fragkiadoulaki I., Alegakis A., Kiriakakis M., Panagiotou N., Ntoumou E., Gratsias I. (2024). Advancing athletic assessment by integrating conventional methods with cutting-edge biomedical technologies for comprehensive performance, wellness, and longevity insights. Front. Sports Act. Living.

[35] Baliou S., Spanakis M., Apetroaei M., Ioannou P., Fragkiadaki P., Fragkiadoulaki I., Renieri E., Vakonaki E., Tzatzarakis M.N., Nosyrev A.E. (2025). The impact of exercise on telomere length dynamics: Molecular mechanisms and implications in athletes (Review). World Acad. Sci. J..

[36] Penggalih M.H.S.T., Sutanto Y.S., Taslim N.A., Syahputra R.A., Hardinsyah H., Tjandrawinata R.R., Nurkolis F. (2025). Precision nutrition in sports science: An opinion on omics-based personalization and athletic outcomes. Front. Nutr..

[37] Samani N.J., van der Harst P. (2008). Biological ageing and cardiovascular disease. Heart.

[38] Nawrot T.S., Staessen J.A., Gardner J.P., Aviv P.A. (2004). Telomere length and possible link to X chromosome. Lancet.

[39] Gardner J.P., Li S., Srinivasan S.R., Chen W., Kimura M., Lu X., Berenson G.S., Aviv A. (2005). Rise in insulin resistance is associated with escalated telomere attrition. Circulation.

[40] Demissie S., Levy D., Benjamin E.J., Cupples L.A., Gardner J.P., Herbert A., Kimura M., Larson M.G., Meigs J.B., Keaney J.F. (2006). Insulin resistance, oxidative stress, hypertension, and leukocyte telomere length in men from the Framingham Heart Study. Aging Cell.

